# African Swine Fever Perception, Risk Factors, and Socioeconomic Disparities Among Smallholder Domestic Pig Farmers in Serengeti, Tanzania

**DOI:** 10.1155/tbed/3922067

**Published:** 2025-08-27

**Authors:** Clara Yona, Mariam R. Makange, Eva Moshiro, Jean N. Hakizimana, Zakile A. Mfumbilwa, Abel S. Lupala, Augustino A. Chengula, Gerald Misinzo

**Affiliations:** ^1^Department of Biosciences, College of Natural and Applied Sciences, Sokoine University of Agriculture, Morogoro, Tanzania; ^2^OR Tambo Africa Research Chair for Viral Epidemics, SACIDS Foundation for One Health, Sokoine University of Agriculture, Morogoro, Tanzania; ^3^Department of Wildlife Management, College of Forestry, Wildlife and Tourism, Sokoine University of Agriculture, Morogoro, Tanzania; ^4^Department of Mathematics and Statistics, College of Natural and Applied Sciences, Sokoine University of Agriculture, Morogoro, Tanzania; ^5^Department of Microbiology, Parasitology and Biotechnology, College of Veterinary Medicine and Biomedical Sciences, Sokoine University of Agriculture, Morogoro, Tanzania

**Keywords:** African swine fever, African swine fever virus, biosecurity, domestic pigs, risk factors, Serengeti National Park, Tanzania

## Abstract

African swine fever (ASF) is a hemorrhagic disease of domestic pigs and wild boars. The ASF virus (ASFV), a sole member of the family Asfarviridae and genus *Asfivirus*, causes this devastating disease. In sub-Saharan Africa, ASFV is maintained through three interlinked cycles: the domestic cycle, the pig-tick cycle, and the sylvatic cycle, which collectively sustain its endemic presence in the region. Interaction between wild and domestic pigs at livestock–wildlife interfaces, particularly in protected areas, poses a significant threat to smallholder farmers. This study aimed to investigate the socioeconomic impact and identify potential risk factors associated with ASF in Serengeti National Park's (SNP's) wildlife management area (WMA) in Tanzania. A cross-sectional study involving 110 domestic pig-keeping households in the five villages of SNP's WMA was carried out. A semistructured questionnaire was used to collect data on risk factors and socioeconomic impact associated with ASF from consenting smallholder farmers. Additionally, the observational approach was used to assess pig production's facilities, behaviors and practices involved in domestic pig production and management. Tissue samples including spleen, lymph nodes, and kidney were also collected from dead domestic pigs belonging to two villages, Nattambiso and Robanda, to confirm the existence of the virus in the study area by using polymerase chain reaction (PCR). Descriptive statistics, univariable, and multivariable logistic analyses were performed to determine risk factors associated with ASF occurrence between October 2021 and April 2022 in the study area. A total of 12 ASF outbreaks affecting 1198 cases that led to 969 domestic pig deaths were reported. The ASFV was confirmed to be positive in all domestic pigs from which tissue samples were collected in the included villages. The case fatality rates (CFRs) from the reported previous ASF incidence varied from 77.5% to 85.2% with an overall CFR of 80.8%. A sum of 163,300,000 Tanzanian Shillings (approximately equivalent to 70,085 USD) loss was recorded in the studied WMA. The major risk factors that correlated with ASF occurrence were encountered ASF previously (odds ratio [OR] = 13.58, 95% confidence interval [CI] = 2.79–87.28), selling pig products with ASF before (OR = 9.43, CI = 1.76–70.02), whilst taking no risk action to prevent loss (OR = 0.14, CI = 0.03–0.63) and swill treatment (OR = 0.10, CI = 0.01–0.54) negatively correlated with ASF. Improving awareness on farm-level biosecurity, husbandry, and management practices is vital to preventing ASF outbreaks and wildlife spillover, safeguarding livestock health, and promoting economic stability in wildlife–livestock–human interfaces.

## 1. Background

African swine fever (ASF) is a hemorrhagic disease of domestic pigs and wild boars caused by the ASF virus (ASFV), a double-stranded DNA (dsDNA) arbovirus [1–3]. The ASFV is the sole member of the family Asfarviridae and genus *Asfivirus* [2, 4]. The ASFV virion is enveloped with an icosahedral symmetry and encapsidated dsDNA genome with varying size between 170 and 194 kilo base pairs (kbps) encoding for about 150–190 open reading frames (ORFs) [2, 4–6]. At the moment, there are 24 ASFV genotypes based on nucleotide variations of a 478-base pair (bp) long fragment at the C-terminal end of the *B646L* gene that encodes for the major p72 capsid protein [7, 8]. Although there have been reports of certain genotypes to exclusively occur in a specific country, ASFV has a transboundary genotypic distribution [9–11]. ASFV has been reported to exist in four cycles, namely, the domestic cycle, the pig–tick cycle, the sylvatic cycle between warthogs (*Phacochoerus africanus*), and soft ticks of the genus *Ornithodoros* and the wild boar habitat cycle [12–14]. The recent epidemiological cycle in Europe which involves the Eurasian wild boars (*Sus scrofa*) as the main reservoir threatens possible ASFV spread to new geographical areas and spillover to domestic pig population [15]. ASFV morbidities and lethalities can reach 100%, making ASF the most global constraint in domestic pig production, food security, and livelihood of smallholder farmers. This is most particularly evident in developing countries where adoption of zoosanitary measures for management and eradication is expensive.

In 1921, Montgomery described a fatal disease in pigs, named East ASF in Kenya (then British East Africa) [1] [16]. Since its first description, ASF has remained endemic in Africa affecting up to 35 African countries including the Indian Ocean Islands of Mauritius and Madagascar [3, 17, 18]. The first excursion of ASFV (Genotype I) outside Africa occurred close to the Lisbon Airport, Portugal, in 1957 and afterwards ASF was reintroduced in 1960 with further spread to the Iberian Peninsula, Europe (Spain, Belgium, France, Italy, Malta, and The Netherlands), Caribbean, and South America (Cuba, Dominican Republic, Brazil, and Haiti) [10, 19, 20]. By the mid-1990s, ASF had been eradicated in all non-African regions by depopulation except Sardinia, Italy, until its official eradication in 2024 [15, 21, 22]. The second incursion of ASFV (Genotype II) in mainland Europe was notified in March 2007 in the seaport of Poti, in the Republic of Georgia [23], with a closest link to viruses circulating in the Eastern and Southern African countries [23, 24]. Rapid spread of ASF was later reported by other countries in the Caucasian region, Russian Federation, and Eastern Europe [23, 25–33]. In August 2018, this highly virulent Genotype II ASFV spread to China and later to other Asian countries including Mongolia, Vietnam, Cambodia, Republic of Korea, Laos, Myanmar, Philippines, Timor-Leste, and Indonesia [34–37]. The introduction and expansion of ASF in Asian countries is argued to be caused by the high domestic pig population and high pork consumption, with a 62% consumption rate of total meat consumption. Human activities such as untreated swill feeding, illegal international transportation and trading of infected pigs/pig products, improper wastes disposal from hotels, ships, and aircrafts have greatly accounted for spread of ASF [4, 23, 34, 36–42]. The transboundary spread of ASF has substantially raised a global concern for global domestic pig industry and food security [18, 43].

ASF is endemic in sub-Saharan Africa, with the presence of all 23 ASFV genotypes (I–XVII and XIX-XXIII). The circulation and maintenance of ASF in the region is through ancient sylvatic cycle and domestic cycle, with and without tick involvement [8, 44–48]. Genotype XVIII, initially designated as the 24th ASFV genotype and isolated from a domestic pig in Namibia, has been retired after it was shown to be a mixed infection of Genotypes I and VIII [46]. The ASF transmission dynamics are linked with biotic, habitat-related, and environmental variables [49]. These factors may include domestic pigs population, wild hosts and soft tick vectors, road density, human population, human activities, and habitat area/characteristics. The distribution and behavior of ASF hosts in the region may be affected by vegetation type and land cover percentage, vegetation index, land use, climate change, or the spread to new environment through host movement of.

The first occurrence of ASF in Tanzania dates back to 1962 with reports of major epidemics in 1987 and 1988 in Mbeya and Arusha/Kilimanjaro regions, respectively [50]. Since 2010, ASF outbreaks have resurged in at least 15 local municipalities of Tanzania with early outbreaks recorded in the southern highlands regions of Mbeya and Iringa and remarkably further spread to other regions of the country [50, 51]. The widespread of occurrence of ASF in different parts of Tanzania with endemic status in the southern highlands has been attributed to human activities and poor biosecurity measures [52]. Activities such as swill feeding, uncontrolled movement of domestic pigs, infected pork products that are fed as swill, and lack of zoosanitary measures have been highlighted to accelerate ASF spread [51, 53–59]. The presence of natural reservoirs has been documented to play role in ASF transmission, particularly from the sylvatic cycle to domestic cycle [19, 60, 61]. Understanding ASF transmission and distribution dynamics will help in deepening knowledge on disease epidemiological courses that might have implications for ASF control and risk management. The aim of this study was to assess ASF knowledge, attitudes, practices, risk factors, and socioeconomic disparities among smallholder domestic pig famers neighboring Serengeti National Park's (SNP's) wildlife management areas (WMAs) in Serengeti district, Tanzania.

## 2. Materials and Methods

### 2.1. Study Area

**Figure 1:**
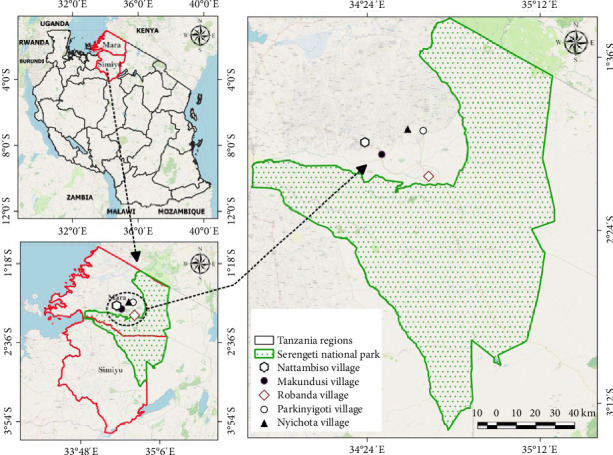
Map of Tanzania showing the study villages of Serengeti National Park's (SNP's) wildlife management area (WMA) in Serengeti district, Tanzania. The map was developed by the authors using QGIS version 3.4.4 [62].

The study was conducted in one of the SNP's WMA in Mara region of Tanzania (Figure 1). The WMA is made up of five villages, namely, Robanda, Nata, Makundusi, Nyinhoka, and Parkinyigoti in Serengeti district, Mara region. The SNP is bordered to the north by the Kenyan border, where it is continuous with the Maasai Mara National Reserve. The SNP is bordered by the Ngorongoro Conservation Area to the southeast, Maswa Game Reserve to the southwest, the Ikorongo and Grumeti Game Reserves to the west, and the Loliondo Game Control Area to the northeast and east. The major socioeconomic activities in this area include tour guiding, crop cultivation, businesses, and livestock keeping. Smallholder farmers in the area practice domestic pig farming mostly for domestic purposes and small businesses to meet their daily household needs. Among other wildlife species, the SNP provides the potential natural habitat for wild pigs (warthogs and bush pigs) and ticks, which are ASFV's sylvatic hosts. The SNP covers an area of 14,750 km^2^ of grassland plains, savanna, riverine forest, and woodlands.

### 2.2. Data Collection

Data collection on knowledge, attitude, practices, risk factors, and socioeconomic impact of ASF in WMA was conducted using a semistructured questionnaire. Questionnaires were administered to 110 consenting smallholder domestic pig farmers living in the study area. Factors including demographic characteristics of respondents, aspects of pig management and husbandry practices, possible transmission factors, ASF risk factors, and socioeconomic impact associated with ASF were collected. Demographic data including gender, age, occupation, annual income, household size, educational, and marital status of respondents were collected. Moreover, ASF awareness, risk factors, attitudes, and practices with respect to pig production and management were assessed. Coordinates of each household involved in the study were recorded using hand-held global positioning system (GPS) receivers.

### 2.3. Data Management and Analysis

Epidemiological data were entered and validated in Excel spreadsheets (Microsoft Corporation, 2023). Data analysis was done using IBM Statistical Package for the Social Sciences (SPSS) software Version 29.0 (IBM Corp., Armonk, NY, USA). Frequencies, percentages, means, and counts were used to determine the distributions and magnitudes of the variables among the interviewed respondents. The level of ASF knowledge, risk perception, and practices were determined using a percentage score out of 100. Association between ASF occurrence and risk factors was determined using logistic regression. A univariate logistic regression was carried out to assess the association between each risk factor with outcome variable (occurrence of ASF in the past 6 months). The analysis was performed using 103 complete cases (excluding missing values), of which 55 of smallholder farmers reported encountering ASF in their current herd and 48 had no ASF cases as for the past 6 months. The risk factors were then ranked based on their *p*-values. Risk factors with smallest *p*-values in univariate analysis were then used in a multivariable logistic regression (Model 1). Several risk factors to be included in the multivariable logistic regression were determined by ratio of frequency of smallest category of outcome variable (ASF occurrence) divided by 10 [63].

Furthermore, as a sensitivity analysis, “grouped lasso” a machine (statistical) learning method was used to automatically select combination of risk factors associated with ASF occurrence [64]. Cross-validation was used to select the combination of risk factors giving the smallest mean square error [65]. The selected combination of risk factors was then used in a multivariate logistic regression (Model 2). Results of Model 2 were compared to that of Model 1. The analysis of association between ASF occurrence and risk factors was performed using R programming language version 4.2.2 [66].

### 2.4. ASFV Diagnosis

Tissue samples including spleen, lymph nodes, and kidney were collected from dead domestic pigs following suspected outbreaks in Nattambiso and Robanda villages. Samples were, thereafter, transported to the Sokoine University of Agriculture (SUA), where virus confirmation was performed. Samples were prepared by chopping 1 g of each sample in a separate sterile petri dish with 5 mL of sterile phosphate-buffered saline (PBS). The homogenized tissues were then centrifuged at 6000 × *g* for 5 minutes at room temperature. Afterwards, the supernatant was transferred into cryovials and stored at −80°C until DNA extraction. Frozen aliquots (100 μL) of preserved tissue supernatant were allowed to thaw and genomic DNA extraction was performed using Qiamp viral nucleic acid extraction kit (Qiagen, Hilden, Germany), according to protocol supplied by the manufacturer. Polymerase chain reaction (PCR) using primers p72U (5′-GGC ACA AGT TCG GAC ATG T-3′)/p72D (5′-GTA CTG TAA CGC AGC ACA G-3′) was performed to amplify the C-terminal region of *B646L* gene which encodes the p72 major capsid protein of the ASFV virion [7].

## 3. Results

### 3.1. Socioeconomic Characteristics of Respondents

In this study, a total of 110 smallholder farmers who are keeping domestic pigs in the study areas were interviewed. Descriptive statistics on socioeconomic characteristics of respondents are given in Table 1. Most of the respondents were male (74%). Respondents' age ranged between 25 and 59 years. The majority of respondents (67%) were married and 41% had attained primary education. About 47% of the respondents had a household size between 1 and 5 people. Both livestock and crop production were reported by 40% of the respondents as primary source of income. However, 59% of respondents mentioned livestock keeping as a secondary source of income. A total of 47 (43%) respondents had an annual income between TZS 1,000,000 and 5,000,000; 38 (34%) between 500,000 and 1,000,000; while the 25 (23%) had an annual income between 100,000 and 500,000. About half of pig owners (51%) claimed to earn between 100,000 and 500,000 from pig production, while the minority 3 (3%) earned below 100,000. At the time this study was conducted the exchange rate for 1 USD was TZS 2330.

### 3.2. General Information and Knowledge

#### 3.2.1. General Farming Information

Table 2 describes the general farming information from the five studied villages. All farmers in four villages (Robanda, Makundusi, Nyichota, and Nattambisso) out of five WMA's villages kept other animals besides domestic pigs. A total of 109 (99%) of the households involved kept other animals for household or food and commercial purposes. About 76 (69%) farmers kept cattle followed by 72 (65%), 69 (63%), and 45 (41%) who kept poultry, sheep, and goat, respectively. Moreover, 56 (51%) farmers kept dogs, for security purposes. About 75% of respondents did not keep health records of their livestock animals. When asked why they do not keep health records, 40% of respondents reported the lack of awareness as a major reason. About 31% of respondents reported “husband and a wife” as the main people responsible for taking care of the herd followed by 26% who reported a “husband” as the main responsible person.

#### 3.2.2. Domestic Pig Husbandry

**Figure 2:**
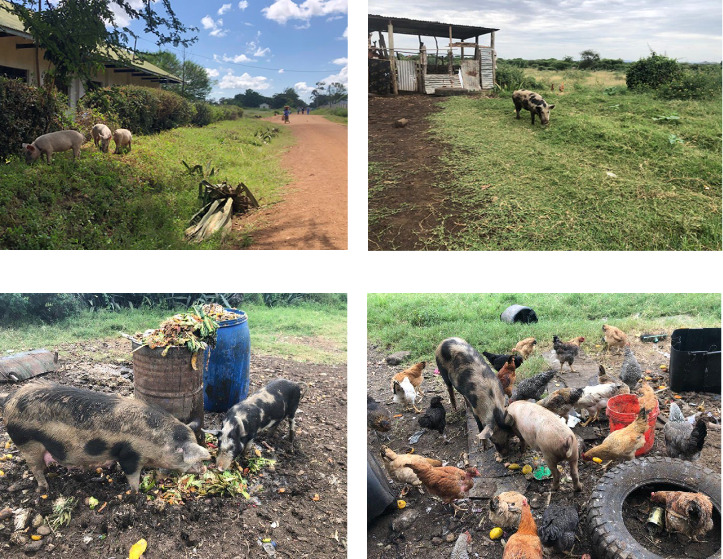
(a, b) Depict freely roaming domestic pigs on streets and near pigpens, as observed in the villages studied. (c, d) Capture freely roaming domestic pigs scavenging on garbage, exposing domestic pigs to uncontrolled waste materials, thus, posing significant biosecurity risks in Nattambiso village, Serengeti district, Tanzania.

Table 2 describes the herd population structure, with most farmers (37%) keeping an average of six to 10 domestic pigs of different age categories. The average number of years that the smallholder farmers practiced domestic pigs keeping was 6 years (Table 2). Meanwhile, on average smallholder farmers reported having the current herd size for about 2.2 years. A total of 918 domestic pigs were recorded during this study survey with 118 (12.8%) adults, 220 (23.9%) growers, 160 (17.4%) weaners, and 420 (45.7%) piglets. The breed types kept by farmers were 70% local breed, 8% exotic breed, and 22% cross breed. The most practiced production system involved both the breeder and grower kept by 76% smallholder farmers. The tethered domestic pig system was mostly practiced by respondents in the studied villages (49%), followed by confined pig management system (27%) and free ranging system (24%). Free ranging domestic pigs were observed in the studied villages (Figure 2). A total of 80% of respondents reported fellow farmers as source of their stock, 14% commercial farmers, and 6% stocked their domestic pigs from nongovernmental organizations (NGOs).

#### 3.2.3. Wild Animals at WMA

Wild pigs, including bushpigs (*Potamochoerus larvatus*) and warthogs (*Phacochoerus africanus*), were reported by 32% of farmers in the studied villages. Other wildlife species occasionally mentioned included wildebeest (*Connochates taurinus*), (*Eguus burchelli*), Thomson's gazelle (*Gazella thomsoni*), buffalo (*Syncerus caffer*), squirrels (*Xerus* spp.), monkeys (*Chlorocebus aethiops*), wild dogs (*Lycaon* spp.) and cats (*Caracal caracal*), foxes (Vulpes spp.), and impala (*Aeopyceros melampus*). Other wild animals reported by farmers included rodents, rats, and snakes. Additionally, 67.3% farmers reported ticks' infestation in domestic pigs and pigpens. Most farmers observed wild animals roaming near their farms and households. Notably, 43% of respondents consumed wild pig meat (Table 3); however, all respondents stated that they had never participated in wild animals hunting or selling wild meat (Table 4).

### 3.3. Domestic Pig Production and Management Practices

#### 3.3.1. Production Practices

Across all WMA's villages, most farmers feed both crop residues and swill (38.2%) to the domestic pig, while some only feed swill or crop residues. Swill (17.3), crop residues supplemented with commercial feeds (11.9%), and commercial feeds alone (9.1%) are the other feeding practices involved (Table 3). The majority of smallholder famers sourced crop residues from their own crops (46.4%) and households and hotel leftovers (40%) were the main sources of swill. Restaurants, hotels, and households were the main source of swill (40%), with 50.9% and 66.3% of respondents reported to serve pig meat that was included in the swill, respectively (Table 3). About 40 (36.4%) smallholder farmers treated swill before feeding their domestic pigs.

Routinely, smallholder farmers treated domestic pigs against parasitic diseases. Approximately 35.5% of smallholder farmers reported using parasite control measures including deworming with albendazole, ivermectin, and levamisole chloride for internal parasites and acaricides spraying for external parasites such as ticks and mites (Table 3). Smallholder farmers reported experiencing poor veterinary services due to the low number of veterinarians and para-veterinarians in the district. Long distance from urban areas and high costs affected the delivery of veterinary services. Farmers only sought consultation with para-veterinarians and veterinarians following serious and prolonged abnormalities and/or disease signs in domestic pigs and not routinely. A total of 50 (45.5%) smallholder farmers relied on local agro–veterinary shops and fellow farmers for drugs, supplements, and farming advice. Most respondents reported diseases as the greatest challenge facing domestic pig production in the studied WMA. Altogether, limited market opportunities and poor access to reliable veterinary services continue to constrain the growth and sustainability of domestic pig farming in the studied WMA.

Grass and timber walls with uncemented floors were the most observed pigpens in all WMA's villages followed by open roof pigpens with similar walls and floor features. Only 22 (20%) smallholder farmers had gates at farms' entrance, 50.9% allowed visitors into pigpens, and footbaths were not available in majority of households (70%; Table 3). Visitors including middlemen, fellow farmers, and neighbors were reported to enter pigpens without any restrictions.

#### 3.3.2. Pig Slaughtering Practices

**Figure 3:**
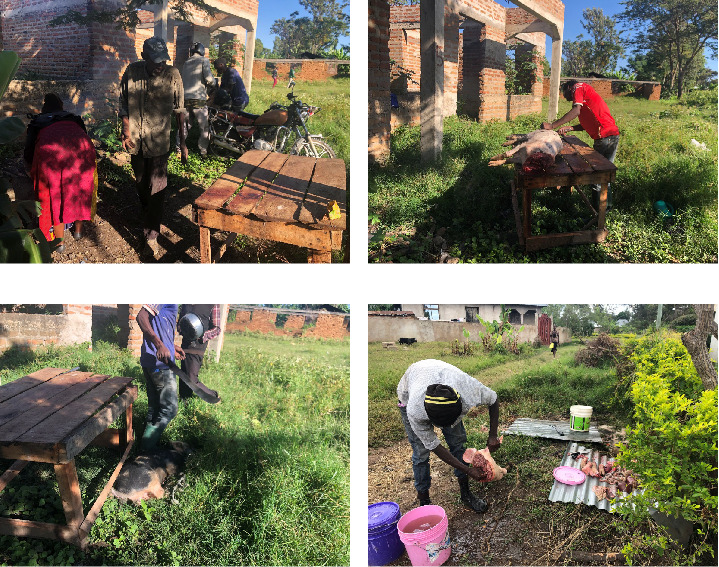
(a, b) Illustrate the delivery and preparation process for the slaughtering activities. (c, d) Depict domestic pig slaughtering practices conducted in homesteads within the studied villages. These activities present potential risk factors for the spread of ASF.

Most smallholder farmers engaged in business purposes, where they either sold domestic pigs or pork to middlemen, traders, butchers, and local pork restaurants. There were no formal slaughter slabs observed in the studied villages. About 68% of farmers slaughtered their domestic pigs on their backyard for households' consumption or business purposes (Figure 3). Poor hygiene and waste management were observed during slaughtering with wastewater left flowing without proper channels to the prepared waste areas. Moreover, offal was fed to livestock including dogs and domestic pigs or thrown away in the local landfill without any account of biosecurity measures. There were reports of scavenging pigs and wild animals in local landfills.

#### 3.3.3. Waste Management Practices

The majority of smallholder farmers (52.7%) cleaned pig pens routinely that involved manure, litter, and other wastes removal, and only 45 (40.9%) farmers used disinfectants as shown in Table 4. Smallholder farmers in this WMA practiced improper waste management with no standard waste disposal pits (Table 4). It was observed that the majority of smallholder farmers threw away domestic pig manure and bedding in their farms or open areas with inclination to contaminate the environment. Only 12 (10.9%) farmers practiced proper waste management including deep burial and burning of carcasses. It was reported that quarantine orders were imposed during ASF outbreaks for the purpose of containing the disease. However, majority of respondents (59.6%) claimed to not adhering to quarantine conditions and instead were engaged in risky practices like domestic pigs selling and slaughtering (Table 4).

### 3.4. Attitudes

When asked whether there are public risks related to ASF, 58% of respondents stated that they believe that there are public risks and 61% of respondents claimed that there are also public health risks with other pig diseases. A total of 50% of respondents were aware of other ASF control methods apart from the ones practiced. Most domestic pigs farmers mentioned treatment and medicine as the main control method, followed by biosecurity measures (13%) and disinfection (11%). Smallholder farmers mentioned swill feeding (25%) and contact among domestic pigs (23%) as potential risk factors for ASF spread and transmission. Lack of awareness (40%) and funds (37%) were reported as the main hindrance in adopting such control practices in the studied WMA. A total of 31% of respondents indicated that training on ASF would improve awareness and could be a proper incentive for the adoption of biosecurity protocols. Smallholder farmers further added the need of financial support from both the government and NGOs to increase the domestic pigs production and livelihoods improvement.

### 3.5. Diseases and ASF Outbreaks in WMA

#### 3.5.1. ASF General Information

The most common diseases/syndrome affecting domestic pigs as experienced were worms, fever, diarrhea, pneumonia, and mange. A total of 45 respondents reported fever as the most fatal disease affecting domestic pig keeping and production in the studied villages, followed by worms (27%; Table 5). A total of 88% of domestic pigs farmers claimed to have heard about ASF. Media was the most reported source of ASF information (48.1%). Famers in the studied WMA's villages mentioned fever (78%) as a local/vernacular name of ASF. A total of 47% of smallholder farmers did not know exactly the main cause of ASF, while 42% and 10% mentioned virus and bacteria, respectively (Table 5). Farmers (69.4%) reported weakness and shivering as the most observed clinical signs of ASF outbreaks. Other reported clinical signs were loss of appetite (18%), redness of the skin (13%), and breathing difficulties (7%). A total of 45% and 49% of domestic pig farmers mentioned adult pigs, piglets, and local breed as the most affected groups and breed, respectively. Among the respondents, 53% claimed that the disease occurs during the wet season and 31% during dry season, while 18% had no idea on which season the disease occurs.

#### 3.5.2. ASF Morbidity and Lethality

A total of 59% of farmers had reported encountering ASF in their current herd between October 2021 and April 2022, with most cases reported in Nattambiso village (14%). About 22 (20%) of smallholder farmers in the studied area reported the suspected ASF to the paraveterinarians. A total of 12 ASF outbreaks were reported in the study villages affecting 1198 domestic pigs, out of which 80% were reported dead. The case fatality rates (CFRs) varied from 77.5% to 85.2% with an overall CFR of 80.8% (Table 6). When asked about actions taken following ASF outbreaks, 33% of interviewed smallholder farmers slaughtered their sick domestic pigs and 25% sold their sick domestic pigs, while 21% treated their sick domestic pigs and disinfected pigpens (Table 6). Makundusi (36%) and Nattambiso (35%) had the highest number of respondents who claimed to slaughter sick domestic pigs following ASF outbreaks as an action to reduce and/or prevent loss. An estimated total loss of 163,300,000 Tanzanian Shillings (approximately equivalent to 70,085 USD) is reported in the studied WMA's villages (Table 7).

#### 3.5.3. Confirmatory Diagnosis

**Figure 4:**
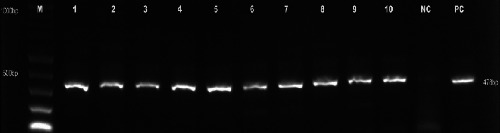
Agarose gel indicating ASFV confirmation in the collected samples with 478 bp expected band size, samples (1–10), control (NC, negative control; PC, ASF positive control), and marker (M).

In total, 10 samples were collected from four farms that encountered ASF in Nattambiso and Robanda villages. All samples collected from the studied area were screened using ASF diagnostic PCR. Analysis of collected tissue samples confirmed the presence of ASFV in all samples (Figure 4). The PCR products of ASFV nucleic acid with a band size of 478 bp using primers p72U and p72D were obtained.

### 3.6. Risk Factors Associated With ASF

The results of the univariate logistic regression are presented in Table 8. Four variables with the lowest *p*-values were identified: prior occurrence of ASF, selling pig products after ASF outbreaks, taking no risk actions to prevent loss, and the number of years of domestic pig keeping (Supporting Information 1: Table 1). These four variables, based on the pseudo-*R*^2^, explained approximately 46% of the variation in the occurrence of ASF in the current herd. The odds of reporting ASF in the current herd were significantly higher among those who had previously experienced ASF, purchased pigs or pig products from ASF-affected sources, and those with more years of experience in domestic pig keeping. In contrast, those who reported to avoid risk practices had lower odds of reporting ASF occurrence (Table 9 and Supporting Information 2: Table 2).

In the sensitivity analysis using “grouped lasso,” Model 2 included 10 variables as risk factors (Table 10; Supporting Information 3: Table 3 and Supporting Information 4: Table 4). In Model 2, similar to Model 1, the variables explained 46% of the variation in the occurrence of ASF in the current herd. Four variables were statistically associated with ASF occurrence in Model 2, three of which were consistent with Model 1: prior encounter with ASF (odds ratio [OR] = 13.58, confidence interval [CI] = 2.79–87.28), selling pig products after previous ASF outbreaks (OR = 9.43, CI = 1.76–70.02), avoiding risk practices (OR = 0.14, CI = 0.03–0.63), and swill treatment (OR = 0.10, CI = 0.01–0.54; Table 9). Although kitchen left over with pig meat was not significant, the *p*-value of its importance to its potential was borderline.

Unlike Model 1, the number of years of domestic pig keeping was not significant in Model 2. However, both avoiding risk practices and swill treatment were significantly associated with reducing the odds of ASF occurrence.

## 4. Discussion

Understanding the behaviors of farmers, traders, butchers, middlemen, and other stakeholders involved in pig production and trade can help in ASF control interventions. Knowledge of practices, socioeconomic factors, and incentives can influence the adoption of disease control measures. Understanding knowledge, attitude, and practices is key towards improvement of a satisfactory level for disease control programs. The current study aimed to evaluate the ASF-related knowledge, attitude, and practices of smallholder farmers, socioeconomic, and risk factors associated with ASF in the studied WMA at SNP, Tanzania. Results of this study can aid veterinarians, researchers, policymakers, government officials, and other stakeholders to adapt their approaches in the studied area for better implementation of the control programs.

Among the smallholder farmers interviewed, the majority (59%) reported experiencing ASF in the past 6 months. Farmers lost between 90% and 100% of their domestic pigs' stocks due to ASF. Financial losses reported by ASF outbreaks in the past 6 months were estimated to be 163,300,000 Tanzanian Shillings due to pig losses, approximately equivalent to 70,085 USD (exchange rate: 1 USD = 2330 TZS). Smallholder farmers performed panic sales and domestic pigs slaughtering during the ASF outbreak to minimize the losses. Although smallholder farmers claimed to recover some funds, such activities might accelerate ASF spread and transmission to neighboring villages and districts [67]. Some smallholder farmers experienced more financial losses in efforts of self-treating their domestic pigs as reported in other places [55]. Moreover, ASF outbreaks in the area have affected smallholder farmers' livelihood stability and growth in terms of loss of food security, medical services, school fees for children, and household expenses. One of the smallholder farmers described his experience in the domestic pig industry:

“*This is more of a betting business, it is true that you get good money, but sometimes we experience devastating losses. Last year I lost over TZS 20 million, I had to sell my house so that I can pay my bank loan*.”

To date, there is no effective vaccine for ASF, which is one of the highest ranked diseases of domestic pigs by morbidity and lethality impact [59]. In consequence, the implementation of biosecurity measures remains the crucial element for ASF management globally. Additionally, poor biosecurity measures in production and management practices can significantly increase ASF spread [52]. In the current study, smallholder farmers poorly implemented biosecurity practices (observation during survey). The sharing of farm equipment and tools can contribute to the occurrence of ASF and facilitate its spread to other areas. Equipment such as shovels, brooms, knives, cutlasses, and wheelbarrows were often shared between backyard abattoirs and farms. Without proper cleaning and disinfection, this practice poses a significant risk to susceptible domestic pigs. Similar findings have been communicated in studies from various regions of Tanzania [52, 55, 57, 59] and Uganda [68, 69]. In Uganda, studies have demonstrated the positive impact of training smallholder farmers on biosecurity measures for effective ASF management and broader public health benefits [70–73]. Globally, ASF control efforts heavily depend on the implementation of strict biosecurity protocols aimed at minimizing the introduction and spread of the disease.

Insufficient knowledge on good husbandry and pig management practices has been reported among constraints facing smallholder farmers in Eastern Africa [70–73]. Although the majority of farmers in the studied WMA acquired primary school education, their education level did not influence their husbandry and slaughtering practices (observation during survey). Nevertheless, farmers with formal education may have a wide opportunity to access knowledge on biosecurity practices based on their background as previously reported by Dione et al. [70]. In the current study, few farmers reported acquiring knowledge on general husbandry and slaughtering practices through training from rural development partners like NGOs.

Experience and informal education have been recognized in various countries, such as Uganda, as valuable contributors to effective husbandry and biosecurity practices [71, 72]. The contribution of informal education, knowledge gained through years of domestic pig keeping such as swill treatment methods naturally accumulates overtime and are closely linked to the length of experience in pig farming. This experiential learning may help explain the observed association between the number of years of domestic pig keeping and reporting ASF, as seen in the univariate analysis (Tables 7 and 8). The sensitivity analysis (Table 9) further supports this by suggesting that hands-on experience and informal knowledge significantly influence farmers' ability to recognize and manage ASF outbreaks. These findings emphasize the need for targeted training programs, particularly for new or less experienced farmers as key players and other stakeholders are equipped with the knowledge and skills necessary for effective ASF prevention, control, and reporting [70, 72]. Additionally, incorporating awareness of emerging and reemerging diseases at the wildlife–livestock–human interface should be integrated into primary education curricula to foster early understanding of disease transmission and prevention, ultimately promoting a more informed and proactive future generation of livestock keepers and community members.

The study observed panic-driven activities by farmers to mitigate losses including premature backyard slaughter and sale, disinfection, and treatment. These activities, while driven by the fear of financial ruin, can sometimes exacerbate the spread of the disease [57, 72]. Quarantine breaching was reported through secret movements of domestic pigs to other locations for trade purposes. Another risky activity was the backyard selling of domestic pigs and pork products at lower prices termed as “panic selling” particularly in sub-Saharan Africa and Asia [70, 73–75]. Purchase and/or selling of pigs during ASF outbreaks is one of risk factors that influenced ASF occurrence in the WMA. The introduction of domestic pigs from fellow farmers during outbreaks could have contributed to the introduction of ASF into farms as a result of the unknown health status of new stock. Moreover, smallholder farmers' tendency of lowering prices during ASF outbreaks that is “panic selling” in the studied WMA accelerated domestic pigs movement. Similar findings have been reported in other parts of Tanzania [57, 67], Uganda [68–70], and Nigeria [76], where movement of domestic pigs were associated with local spread between and within farms. Furthermore, interdistrict domestic pigs movements for trade purposes were associated with ASFV spread in Tanzania and Uganda–Kenya border [57, 72]. In Uganda, Dione et al. [70] reported the fear of losing domestic pigs as there is no compensation scheme offered by the government. Efforts must, therefore, be made to reduce uncontrolled local and interdistrict domestic pigs selling during ASF outbreaks to prevent further spread into new areas.

In contrast, taking no action, including the absence of panic-driven activities, was found to have a negative correlation with the occurrence of ASF and spread in the studied WMA. However, the study did not analyze whether a lack of proactive activities was from the awareness of risk factors or not. Thus, the study underscores the critical importance of raising awareness among pig value chain actors on timely reporting of ASF to authorities which will result in proper post-ASF actions. Such reporting enables swift and effective disease detection and investigation for effective management and control efforts by veterinarians and public health officials [71]. Lack of awareness, limited access to veterinary services, and fruitful assistance by authorities in the event of an outbreak have accounted for underreporting of ASF [77, 78]. Additionally, fear of unfavorable consequences as perceived by pig value chain actors such as quarantine, pig movement bans, culling infected pigs, and the absence of compensation programs discourage farmers from reporting outbreaks [70, 77, 79, 80]. However, without such immediate containment measures, ASF can rapidly spread through direct contact with infected domestic pigs, contaminated feed or even through people and vehicles moving between farms, districts, and countries [72, 73, 77].

Swill feeding was one of the feeding strategies used by smallholder farmers in the studied villages. Untreated or undercooked swill increases the risk of the introduction of ASFV into farms. Swill feeding has mostly been recorded in the urban and peri-urban areas due to their proximity to food services centers. However, due to unavailability and poor accessibility of good commercial feeds, swill feeding was highly practiced by smallholder farmers in the studied areas [72]. Households, hotels, and restaurants located in the SNP and surrounding villages were reported as the main sources of swill. In this study, treatment of swill was found to significantly reduce the odds of ASF occurrence in the studied areas. Similar to previous studies, the study agrees that poor feeding practices including the use of undercooked swill and contaminated food and drinking water play a role in the emergence and spread of ASF [55, 72, 76, 81]. Emphasis on proper planned feed stores, covered drinking water storage, and swill treatment must be incorporated in farms' biosecurity protocols.

In Eastern Africa, the involvement of wild pigs (commonly warthogs and bush pigs) which act as wild reservoir hosts play a role in the existence of ASFV in the region through the sylvatic cycle involving *Ornithodoros* ticks [11, 16, 58, 82–85]. The detection of ASFV in ticks and ASFV specific antibodies in warthogs confirms the persistence of sylvatic cycle in the region and raises concerns about the potential risk of spilling over to domestic pigs [58]. Furthermore, studies such as Katale et al. [82] in the Serengeti ecosystem suggest that free-ranging domestic pig production systems near protected areas increase the likelihood of tick mediated transmission by facilitating overlapping habitats [58]. Measures including effective separation between domestic pigs and wild hosts and/or products and modernization of husbandry systems which has proven to improve management in sub-Saharan Africa are encouraged within this WMA [86]. Furthermore, future studies should prioritize comprehensive surveillance of wild host populations and their interactions with domestic pigs, alongside molecular characterization of circulating ASFV strains. Such integrated approaches will enhance understanding of transmission dynamics and support targeted interventions within this WMA.

*Ornithodoros* soft ticks, also known as tampans are invertebrate hosts and the only known arthropod biological vectors of ASFV [11, 16, 19, 84]. Although the presence of *Ornithodoros* ticks in domestic pig premises has been documented across various ASF endemic regions including parts of Southern Africa, the Iberian Peninsula, and Madagascar, the domestic pig–tick cycle remains insufficiently investigated. The few studies conducted have shown the ability of infected *Ornithodoros* ticks to retain the virus for long periods even in the absence of blood meals, highlighting their role in ASFV maintenance and persistence particularly in settings with poor pigsty infrastructure that favors tick survival [11, 44, 87]. The *O. moubata* ticks are widely distributed in Eastern Africa and have been reported in the warthogs' burrows and pigpens [83, 84, 88, 89]. The ASFV transmission from infected ticks to domestic pigs occur through a tick bite [11, 19]. Genetically similar ASFV strains have been identified in both warthog-associated *Ornithodoros* ticks and domestic pig outbreaks [58, 82]. Altogether poor domestic pigpens and free roaming system in the studied WMA create a favorable environment for tick infestation as reported in previous studies [19, 90].

## 5. Conclusion

The findings from this study highlight the challenges in domestic pigs production, management, and risk factors in the SNP WMA. ASF has a devastating effect on the livelihoods of local communities reliant on pig farming, threatening food security, and leading to significant economic losses. Understanding ASF dynamics is vital to implementing preventive measures that ensure the health of both livestock and wildlife in this ecological and economically critical region. Further studies can significantly improve potential interventions for the improvement of biosecurity efforts for ASF control. When managing the WMA, focus should also be placed on enhancing government policy to ensure proper production standards and compensatory measures that boost productivity and sustain the domestic pig industry, especially at wildlife–livestock–human interfaces.

## Figures and Tables

**Table 1:** Socioeconomic characteristics of respondents engaged in the studied wildlife management area.

Category	Variable	Robanda	Parkinyigoti	Nattambisso	Makundusi	Nyinchoka	Total
		*n* (%)	*n* (%)	*n* (%)	*n* (%)	*n* (%)	*n* (%)
Sex	Male	16 (84.2)	15 (62.5)	12 (60)	19 (86.4)	19 (76)	81 (74)
	Female	3 (15.8)	9 (37.5)	8 (40)	3 (13.6)	6 (24)	29 (26)
	Total	19 (100)	24 (100)	20 (100)	22 (100)	25 (100)	110 (100)

Age	Mean	43.9	38.0	34.3	40.9	41.4	40
	Std. deviation	9.3	10.1	8.7	7.8	4.5	9
	Minimum	25.0	23.0	19.0	28.0	33.0	19
	Maximum	59.0	59.0	50.0	59.0	56.0	59

Marital status	Single	1 (5.3)	2 (8.3)	3 (15)	3 (13.6)	4 (16)	13 (12)
	Married	18 (94.7)	18 (75)	10 (50)	13 (59.1)	15 (60)	74 (67)
	Widow	0 (0)	3 (12.5)	2 (10)	1 (4.5)	3 (12)	9 (8)
	Separated	0 (0)	1 (4.2)	4 (20)	3 (13.6)	3 (12)	11 (10)
	Divorced	0 (0)	0 (0)	1 (5)	2 (9.1)	0 (0)	3 (3)

Education level	No formal education	3 (16)	7 (29)	9 (45)	3 (14)	5 (20)	27 (25)
	Primary level	13 (68)	11 (46)	1 (5)	10 (46)	10 (40)	45 (41)
	Secondary level	3 (16)	6 (25)	6 (30)	8 (36)	9 (36)	32 (29)
	College/university	0 (0	0 (0)	4 (20)	1 (5)	1 (4)	6 (5)

Household size	1–5	9 (47)	14 (58)	11 (55)	7 (31.8)	11 (44)	52 (47)
	6–10	10 (52.6)	10 (42)	9 (45)	13 (59.1)	7 (28)	49 (45)
	11–15	0 (0)	0 (0)	0 (0)	2 (9.1)	7 (28)	9 (8)

Primary source of income	Pastoralism	9 (47.4)	8 (33.3)	7 (35)	10 (45.5)	10 (40)	46 (42)
	Agriculture	6 (31.6)	9 (37.5)	9 (45)	10 (45.5)	10 (40)	44 (40)
	Business	3 (15.8)	6 (25)	4 (20)	2 (9.1)	5 (20)	20 (18)
	Researcher	1 (5.3)	1 (4.2)	0 (0)	0 (0)	0 (0)	0 (0)

Secondary source of income	Pastoralism	10 (52.6)	15 (62.5)	13 (65)	12 (54.5)	15 (60)	65 (59)
	Fishing	0 (0)	0 (0)	0 (0)	0 (0)	0 (0)	0 (0)
	Agriculture	8 (42.1)	1 (4.2)	4 (20)	10 (45.5)	10 (40)	41 (37)
	Business	1 (5.3)	8 (33.3)	3 (15)	0 (0)	0 (0)	4 (4)

Annual income (TZS)	100,000–500,000	3 (15.8)	10 (41.7)	5 (25)	2 (9.1)	5 (20)	25 (23)
	500,000–1,000,000	6 (31.6)	6 (25)	13 (65)	9 (40.9)	4 (16)	38 (34)
	1,000,000–5,000,000	10 (52.6)	8 (33.3)	2 (10)	11 (50)	16 (64)	47 (43)

Income from pig production (TZS)	<100,000	0 (0)	1 (4.2)	0 (0)	0 (0)	2 (8)	3 (3)
	100,000–500,000	7 (36.8)	14 (58.3)	16 (80)	8 (36.4)	11 (44)	56 (51)
	500,000–1,000,000	10 (52.6)	7 (29.2)	2 (10)	12 (54.5)	10 (40)	41 (37)
	1,000,000–5,000,000	2 (10.5)	2 (8.3)	2 (10)	2 (9.1)	2 (8)	10 (9)

Activities in the pig value chain	Middleman	0 (0)	0 (0)	0 (0)	0 (0)	0 (0)	0 (0)
	Pork seller	0 (0)	0 (0)	0 (0)	0 (0)	0 (0)	0 (0)
	Owner of pigs	19 (100)	24 (100)	20 (100)	22 (100)	25 (100)	110 (100)

Abbreviation: TZS, Tanzanian Shillings.

**Table 2:** General domestic pig husbandry practices in the studied wildlife management area.

Category	Variable	Robanda	Parkinyigoti	Nattambiso	Makundusi	Nyinchoka	Total
		*n* (%)	*n* (%)	*n* (%)	*n* (%)	*n* (%)	*n* (%)
Source of stock	Fellow farmers	19 (100)	17 (71)	18 (90)	16 (73)	18 (72)	88 (80)
	Commercial farmers	0 (0)	2 (8)	0 (0)	6 (27)	7 (28)	15 (14)
	NGOs	0 (0)	5 (21)	2 (10)	0 (0)	0 (0)	7 (6)
	Total	19 (100)	24 (100)	20 (100)	22 (100)	25 (100)	110 (100)

Breed type	Local breed	19 (100)	17 (71)	12 (60)	14 (64)	15 (60)	77 (70)
	Exotic breed	0 (0)	1 (4)	2 (10)	2 (9)	2 (8)	9 (8)
	Cross breed	0 (0)	6 (25)	6 (30)	6 (27)	8 (32)	24 (22)

Years practiced pig farming	1–5	6 (32)	11 (46)	7 (35)	5 (23)	10 (40)	39 (36)
	6–10	5 (26)	9 (38)	8 (40)	10 (46)	9 (36)	41 (37)
	11–20	5 (26)	3 (13)	3 (15)	7 (32)	6 (24)	24 (22)
	>20	3 (16)	1 (4)	2 (10)	0 (0)	0 (0)	6 (6)

Production system	Only breeder	0 (0)	5 (21)	6 (30)	0 (0)	0 (0)	11 (11)
	Only grower	2 (11)	7 (29)	5 (25)	0 (0)	1 (4)	15 (14)
	Breeder and grower	17 (90)	12 (50)	9 (45)	22 (100)	24 (96)	84 (76)

Pig management system	Free ranging system	1 (5)	2 (8)	7 (35)	5 (23)	10 (40)	25 (23)
	Tethered system	13 (68)	12 (50)	6 (30)	13 (59)	9 (36)	53 (48)
	Confined system	5 (26)	10 (42)	7 (35)	4 (18)	6 (24)	32 (29)

Responsible person	Husband	6 (32)	4 (17)	5 (25)	6 (27)	5 (20)	26 (24)
	Wife	1 (5)	2 (8)	2 (10)	1 (5)	6 (24)	12 (11)
	Children	0 (0)	3 (13)	5 (25)	1 (5)	1 (4)	10 (9)
	Both 1 and 2	7 (37)	8 (33)	3 (15)	9 (41)	7 (28)	34 (31)
	Both 1, 2, and 3	1 (5)	2 (8)	0 (0)	4 (18)	3 (12)	10 (9)
	Hired labor	1 (5)	4 (17)	4 (20)	0 (0)	0 (0)	9 (8)
	1 and 3	2 (11)	1 (4)	0 (0)	1 (5)	2 (8)	6 (6)
	Husband, wife, and hired labor	1 (5)	0 (0)	1 (5)	0 (0)	1 (4)	3 (3)

Other animals	Yes	19 (100)	23 (96)	20 (100)	22 (100)	25 (100)	109 (99)
	No	0 (0)	1 (4)	0 (0)	0 (0)	0 (0)	1 (1)

**Table 3:** Domestic pig production and management practices in the studied villages, Serengeti, Tanzania.

Category	Variable	Robanda	Parkinyigoti	Nattambiso	Makundusi	Nyichota	Total
		*n* (%)	*n* (%)	*n* (%)	*n* (%)	*n* (%)	*n* (%)
Pig feeding practices	Crop residues + commercial feeds	1 (5)	2 (8)	5 (25)	2 (9)	2 (8)	12 (10.9)
	Crop residues + swill	11 (58)	7 (29)	6 (30)	8 (36)	10 (40)	42 (38.2)
	Crop residues	1 (5)	2 (8)	1 (5)	3 (14)	1 (4)	8 (7.3)
	Swill alone	4 (21)	6 (25)	2 (10)	5 (23)	2 (8)	19 (17.3)
	Pastures	0 (0)	1 (4)	1 (5)	1 (5)	5 (20)	8 (7)
	Commercial feeds	1 (5)	2 (8)	3 (15)	1 (5)	3 (12)	10 (9)
	Crop residues + pasture	1 (5)	4 (17)	2 (10)	2 (9)	2 (8)	11 (10)
	—	19 (100)	24 (100)	20 (100)	22 (100)	25 (100)	29 (26)

Swill feeding	Yes	11 (58)	12 (50)	15 (75)	11 (50)	12 (48)	61 (55)
	No	8 (42)	12 (50)	5 (25)	11 (50)	13 (52)	49 (45)

Source of crop residues	Own plantationcrops	10 (53)	13 (54)	7 (35)	8 (36)	13 (52)	51 (46)
	Own plantationcrops and fellow farmers	5 (26)	6 (25)	9 (45)	9 (41)	8 (32)	37 (34)
	Fellow farmers	4 (21)	5 (21)	4 (20)	5 (23)	4 (16)	22 (20)

Source of swills	Hotel	2 (11)	5 (21)	7 (35)	4 (18)	6 (24)	24 (22)
	Own households	4 (21)	6 (25)	3 (15)	6 (27)	6 (24)	25 (23)
	Neighbor households	2 (11)	3 (13)	3 (15)	4 (18)	5 (20)	17 (15)
	Hotel + households	11 (58)	10 (42)	7 (35)	8 (36)	8 (32)	44 (40)

Pig meat in household leftovers	Yes	11 (58)	15 (63)	14 (70)	11 (50)	5 (20)	56 (51)
	No	8 (42)	9 (38)	6 (30)	11 (50)	20 (80)	54 (49)

Pig meat in hotel leftovers	Yes	16 (84)	11 (46)	9 (45)	18 (82)	19 (76)	73 (66)
	No	3 (16)	13 (54)	11 (55)	4 (18)	6 (24)	37 (34)

Pigs diseases prevention practices	Parasite control	5 (26)	9 (38)	8 (40)	7 (32)	10 (40)	39 (35)
	Iron injection	3 (16)	5 (21)	6 (30)	5 (23)	5 (20)	24 (22)
	Vitamins	3 (16)	2 (8)	5 (25)	2 (9)	3 (12)	15 (14)
	Parasite control and vitamins	4 (21)	5 (21)	1 (5)	5 (23)	5 (20)	20 (18)
	None	4 (21)	3 (13)	0 (0)	3 (14)	2 (8)	12 (11)

Common dewormers	Acaricides spraying	1 (5)	7 (29)	4 (20)	6 (27)	4 (16)	22 (20)
	Ivermectin	3 (16)	3 (13)	2 (10)	2 (9)	4 (16)	14 (13)
	Levamisole chloride	3 (16)	2 (8)	1 (5)	3 (14)	3 (12)	12 (11)
	Albendazole	10 (53)	7 (29)	7 (35)	5 (23)	8 (32)	37 (34)
	Others	2 (11)	5 (21)	6 (30)	6 (27)	6 (24)	25 (23)

Source of treatment	Fellow farmers	5 (26)	6 (25)	5 (25)	3 (14)	6 (24)	25 (23)
	Para-veterinarian	4 (21)	6 (25)	3 (15)	4 (18)	4 (16)	21 (19)
	Veterinarian	2 (11)	2 (8)	3 (15)	4 (18)	2 (8)	13 (12)
	Agro–vet shop	7 (37)	10 (42)	9 (45)	11 (50)	13 (52)	50 (45)
	None	1 (5)	0 (0)	0 (0)	0 (0)	0 (0)	1 (1)

Pig introduction in the past 6 months	Yes	12 (63)	15 (63)	12 (60)	12 (55)	14 (56)	65 (59)
	No	7 (37)	9 (38)	8 (40)	10 (45)	11 (44)	45 (41)

Slaughtering	Home backyard	16 (84)	19 (79)	16 (80)	15 (68)	9 (36)	75 (68)
	Slaughter slabs	3 (16)	5 (21)	4 (20)	7 (32)	16 (64)	35 (32)

Wild pig hunting	Yes	9 (47)	7 (29)	5 (25)	11 (50)	15 (60)	47 (43)
	No	10 (53)	17 (71)	15 (75)	11 (50)	10 (40)	63 (57)

Wild pig access to local landfill	Yes	10 (53)	17 (71)	15 (75)	11 (50)	10 (40)	63 (57)
	No	9 (47)	7 (29)	5 (25)	11 (50)	15 (60)	47 (43)

Challenges facing production	Veterinary services	1 (5)	3 (13)	3 (15)	5 (23)	2 (8)	14 (13)
	Market	2 (11)	5 (21)	3 (15)	2 (9)	3 (12)	15 (14)
	Funds	1 (5)	1 (4)	3 (15)	1 (5)	3 (12)	9 (8)
	Production awareness	3 (16)	3 (13)	1 (5)	2 (9)	1 (4)	10 (9)
	Feeds availability	2 (11)	2 (8)	1 (5)	1 (5)	2 (8)	8 (7)
	Diseases	9 (47)	7 (29)	7 (35)	9 (41)	10 (40)	42 (38)
	Drugs availability	1 (5)	3 (13)	2 (10)	2 (9)	4 (16)	12 (11)

Action after ASF	No action	2 (11)	2 (8)	5 (25)	2 (9)	2 (8)	13 (12)
	Slaughtered	5 (26)	6 (25)	7 (35)	8 (36)	7 (28)	33 (30)
	Sold	6 (32)	7 (29)	5 (25)	7 (32)	12 (48)	37 (34)
	Treatment	4 (21)	8 (33)	1 (5)	5 (23)	2 (8)	20 (18)
	Disinfection	2 (11)	1 (4)	2 (10)	0 (0)	2 (8)	7 (6)

**Table 4:** List of biosecurity practices by smallholder farmers in the studied villages.

Variable	Yes	No	Percentage compliance (%)
Routine cleaning of pigpens	58	52	52.7
Equipment and tools sharing	30	80	27.3
Equipment and tools washing with disinfectant	25	85	22.7
Keeping health records	27	83	24.5
Swill treatment	40	70	36.4
Use of disinfectants	45	65	40.9
Use of safety protective farm gears	52	58	47.3
Regular changing of farm clothes	13	97	11.8
Gate at farm's entrance	22	88	20.0
Allowing visitors into pigpens	56	54	50.9
Foot bath at farm's entrance	33	77	30.0
Regular changing of foot bath	15	95	13.6
Quarantine of new stock	10	100	9.1
Health status of new stock	8	102	7.3
Food and water control	15	95	13.6
Ticks in pigs/pigpens	74	36	67.3
Rodent and livestock access to pigpens	78	32	70.9
Keeping pigs according to age	24	86	21.8
Keeping pigs according to species	22	88	20.0
Space between pigs	23	87	20.9
Safe waste disposal	15	95	13.6
Burying of intestinal content after slaughter	12	98	10.9
Scavenger access to pig farms	88	22	80.0
Sick pigs' isolation	5	105	4.5
Prompt disposal of dead pigs	78	32	70.9
Regular consultation with veterinarians	23	87	20.9
Contact with infected farms	29	81	26.4
Wild pig hunting	0	110	100
Wild pigs access to local landfill	63	47	110

*Note:* Yes, percentage compliance group.

**Table 5:** General information regarding domestic pig production and management and ASF in the studied villages, Serengeti, Tanzania.

Category	Variable	Robanda	Parkinyigoti	Nattambiso	Makundusi	Nyichota	Total
		*n* (%)	*n* (%)	*n* (%)	*n* (%)	*n* (%)	*n* (%)
What is the most fatal disease	Worms	6 (32)	10 (42)	13 (65)	0 (0)	1 (4)	30 (27)
	Fever	10 (53)	12 (50)	7 (35)	8 (36)	8 (32)	45 (41)
	Pneumonia	2 (11)	0 (0)	0 (0)	8 (36)	6 (24)	16 (15)
	Others (diarrhea and mange)	1 (5)	2 (8)	0 (0)	6 (27)	10 (40)	19 (17)

Total	—	19 (100)	24 (100)	20 (100)	22 (100)	25 (100)	110 (100)

Heard about ASF before	Yes	17 (89)	22 (92)	15 (75)	20 (91)	23 (92)	97 (88)
	No	2 (11)	2 (8)	5 (25)	2 (9)	2 (8)	13 (12)

Know the cause of ASF	Yes	5 (26)	16 (67)	17 (85)	8 (36)	12 (48)	58 (53)
	No	14 (74)	8 (33)	3 (15)	14 (64)	13 (52)	52 (47)

Cause of ASF	Bacteria	1 (5)	2 (8)	4 (20)	2 (9)	2 (8)	11 (10)
	Do not know	14 (74)	7 (29)	3 (15)	14 (64)	14 (56)	52 (47)
	Virus	4 (21)	15 (63)	13 (65)	6 (27)	9 (36)	47 (43)

Recognize ASF	Yes	13 (68)	17 (71)	14 (70)	11 (50)	13 (52)	68 (62)
	No	6 (32)	7 (29)	6 (30)	11 (50)	12 (48)	42 (38)

ASF common name	Do not know	0 (0)	0 (0)	0 (0)	1 (5)	2 (8)	3 (3)
	Fever	16 (84)	23 (96)	19 (95)	14 (64)	14 (56)	86 (78)
	Pneumonia	3 (16)	1 (4)	1 (5)	7 (32)	9 (36)	21 (19)

Affected age group	Adult boar/sow	4 (21)	6 (25)	5 (25)	7 (32)	9 (36)	31 (28)
	Growers	1 (5)	2 (8)	3 (15)	0 (0)	1 (4)	7 (6)
	Weaners	0 (0)	1 (4)	2 (10)	0 (0)	0 (0)	3 (3)
	Piglets	1 (5)	0 (0)	0 (0)	5 (23)	5 (20)	11 (10)
	1 and 4	8 (42)	12 (50)	10 (50)	10 (45)	10 (40)	50 (45)
	All groups	5 (26)	3 (13)	0 (0)	0 (0)	0 (0)	8 (7)

Affected breeds type	Local breed	10 (53)	11 (46)	6 (30)	14 (64)	13 (52)	54 (49)
	Improved breed	1 (5)	4 (17)	7 (35)	7 (32)	10 (40)	29 (26)
	Do not know	8 (42)	9 (38)	7 (35)	1 (5)	2 (8)	27 (25)

**Table 6:** Epidemiological information of the previous suspected ASF outbreaks in the studied villages.

Location	Outbreak month	Age group affected	Breed affected	Herd size					Morbidity					Lethality					Apparent case fatality rate
				Adults	Weaners	Growers	Piglets	Total	Adults	Weaners	Growers	Piglets	Total	Adults	Weaners	Growers	Piglets	Total	
Robanda	November 2021	All groups	All breeds	59	36	23	51	169	20	12	10	35	77	8	18	12	22	60	77.9
Nattambiso	January– March 2022	All groups	All breeds	134	67	61	97	359	89	62	74	92	317	70	62	56	77	265	83.6
Makundusi	October– December 2021	All groups	All breeds	43	54	62	69	228	33	48	58	88	227	28	45	45	58	176	77.5
Nyichota	February–April 2022	All groups	All breeds	95	76	116	281	568	46	39	75	174	334	28	32	62	139	261	78.1
Parkinyigoti	October–November 2021	All groups	All breeds	77	75	134	147	433	38	37	83	85	243	26	30	66	85	207	85.2
Total	—	—	—	—	—	—	—	1757	—	—	—	—	1198	—	—	—	—	969	—

**Table 7:** Loss estimation following ASF outbreaks in the studied villages.

Age group	Weight (kg)	Number of dead animals	Price (TZS)	Total loss (TZS)
Adults	60–130	160	370,000	59,200,000
Growers	25–60	187	180,000	33,660,000
Weaners	20–45	241	150,000	36,150,000
Piglets	15–25	381	90,000	34,290,000
Total	—	969	—	163,300,000

Abbreviation: TZS, Tanzanian Shillings.

**Table 8:** Univariate logistic regression for risk factors associated with ASF outbreaks in the studied WMA's villages, Serengeti, Tanzania.

Variable	OR	95% CI of OR	*p*-Value
Encounter ASF before	19.46	6.62–72.41	<0.0001
Sold pig product ASF before	7.50	3.07–20.10	<0.0001
Prevent loss no action	0.16	0.06–0.40	<0.0001
Years domestic pig keeping	1.26	1.11–1.45	0.0001
Ticks pigs premises	4.89	2.06–12.34	0.0002
Years of the current herd	1.60	1.16–2.40	0.0014
No ASF control used	0.11	0.02–0.45	0.0024
Treatment for ASF control	0.53	0.13–1.72	0.0024
Swills treatment	0.29	0.12–0.65	0.0027
Aware of control measure	3.24	1.46–7.42	0.0036
Presence of slaughter slabs	3.35	1.31–9.39	0.0106

**Table 9:** Coefficient estimates of association Model 1 (manual selection) using multiple logistic regression to assess risk factors associated with occurrence of ASF.

Variable	OR	95% CI of OR	*p*-Value
Encounter ASF before	9.54	2.49–43.53	0.0017
Sold pig product ASF before	6.29	1.82–24.23	0.0048
Prevent loss no action	0.11	0.02–0.39	0.0011
Years domestic pig keeping	1.31	1.11–1.59	0.0032

**Table 10:** Coefficient estimates of association Model 2 (grouped lasso) using multiple logistic regression to assess risk factors associated with occurrence of ASF.

Variable	OR	95% CI of OR	*p*-Value
Encounter ASF before	13.58	2.75–87.28	0.0025
Sold pig product ASF before	9.43	1.76–70.02	0.0150
Prevent loss no action	0.14	0.03–0.63	0.0147
Years domestic pig keeping	1.19	0.93–1.57	0.1794
Swills treatment	0.10	0.01–0.54	0.0129
Years current herd	1.59	0.88–3.39	0.1655
Aware control measure	3.50	0.75–19.17	0.1193
Protective gear	1.00	0.19–5.07	0.9990
Kitchen leftover pig meat	4.26	0.94–24.05	0.0729
Presence of ticks in pigs premises	4.06	0.70–30.35	0.1349

## Data Availability

The data that support the findings of this study are available upon request from the corresponding author. The data are not publicly available due to privacy or ethical restrictions.

## Nomenclature

ASF: African swine fever
ASFV: African swine fever virus
bp: Base pair
CI: Confidence interval
CO_2_: Carbon dioxide
COSTECH: Tanzania Commission for Science and Technology
DNA: Deoxyribonucleic acid
DPRTC: Directorate of Postgraduate Studies, Research, Technology Transfer and Consultancy
dsDNA: Double-stranded DNA
GPS: Global positioning system
NGOs: Nongovernmental organization
OR: Odds ratio
ORF: Open reading frame
PCR: Polymerase chain reaction
AREC: Animal Research Ethics Committee
SNP: Serengeti National Park
SPSS: Statistical Package for the Social Sciences
SUA: Sokoine University of Agriculture
SUARIS: Sokoine University of Agriculture Research and Innovation Support
TANAPA: Tanzania National Parks
TAWIRI: Tanzania Wildlife Research Institute
VIF: Variance inflation factor
WMA: Wildlife management area.

## Supporting Information

Supporting Information 1 Table 1: Coefficient estimates of the association model.

Supporting Information 2 Table 2: Multicollinearity test of Model 1 showing the variance inflation factor (VIF) for each variable.

Supporting Information 3 Table 3: Regression coefficient for Model 2 variable selected by lasso.

Supporting Information 4 Table 4: Multicollinearity test of Model 2 showing the variance inflation factor (VIF) for each variable.
