# Supplementary material for: African Swine Fever Perception, Risk Factors, and Socioeconomic Disparities Among Smallholder Domestic Pig Farmers in Serengeti, Tanzania
Source: Transbound Emerg Dis. 2025 Aug 27;2025:3922067. doi: 10.1155/tbed/3922067 (PMC12408130; doi:10.1155/tbed/3922067)
Supplement: Supporting Information 1 — Table 1: Coefficient estimates of the association model. [file 3922067.f1.docx]

**Table 1:** Coefficient Estimates of Association Model

| **Term** | **estimate** | **std.error** | **statistic** | **p-value** | **or** | **or_l95** | **or_u95** |
| --- | --- | --- | --- | --- | --- | --- | --- |
| ASF encounter before | 2.2559 | 0.7171 | 3.1459 | 0.0017 | 9.5440 | 2.4948 | 43.5299 |
| Sold pig product with ASF before | 1.8388 | 0.6518 | 2.8211 | 0.0048 | 6.2889 | 1.8220 | 24.2350 |
| No action to prevent loss | -2.1854 | 0.6684 | -3.2695 | 0.0011 | 0.1124 | 0.0277 | 0.3921 |
| Years of domestic pig keeping | 0.2675 | 0.0908 | 2.9443 | 0.0032 | 1.3067 | 1.1071 | 1.5877 |
