# Supplementary material for: African Swine Fever Perception, Risk Factors, and Socioeconomic Disparities Among Smallholder Domestic Pig Farmers in Serengeti, Tanzania
Source: Transbound Emerg Dis. 2025 Aug 27;2025:3922067. doi: 10.1155/tbed/3922067 (PMC12408130; doi:10.1155/tbed/3922067)
Supplement: Supporting Information 2 — Table 2: Multicollinearity test of Model 1 showing the variance inflation factor (VIF) for each variable. [file 3922067.f2.docx]

**Table 2:** Malticolinearity Test of Model 1 showing the Variance Inflation Factor (VIF) for each variable

| **Variable** | **VIF** |
| --- | --- |
| asf_encounter_before | 1.0824 |
| sold_pig_product_asf_before | 1.2412 |
| prevent_loss_no_action | 1.0910 |
| years_domestic_pig_keeping | 1.2355 |

*Based on VIF, the model doesn’t have problem with the multicolinearity with all VIF less than 5 (James et al., 2023).
