# Supplementary material for: African Swine Fever Perception, Risk Factors, and Socioeconomic Disparities Among Smallholder Domestic Pig Farmers in Serengeti, Tanzania
Source: Transbound Emerg Dis. 2025 Aug 27;2025:3922067. doi: 10.1155/tbed/3922067 (PMC12408130; doi:10.1155/tbed/3922067)
Supplement: Supporting Information 3 — Table 3: Regression coefficient for Model 2 variable selected by lasso. [file 3922067.f3.docx]

**Table 3:** Regression Coefficient for Model 2 Variable Selected by LASSO

| **variable** | **estimate** | **std.error** | **statistic** | **p.value** | **or** | **or_l95** | **or_up95** |
| --- | --- | --- | --- | --- | --- | --- | --- |
| asf_encounter_before | 2.6084 | 0.8622 | 3.0254 | 0.0025 | 13.5773 | 2.7521 | 87.2776 |
| sold_pig_product_asf_before | 2.2446 | 0.9228 | 2.4325 | 0.0150 | 9.4370 | 1.7552 | 70.0202 |
| prevent_loss_no_action | -1.9458 | 0.7976 | -2.4397 | 0.0147 | 0.1429 | 0.0262 | 0.6312 |
| years_domestic_pig_keeping | 0.1758 | 0.1309 | 1.3427 | 0.1794 | 1.1922 | 0.9331 | 1.5745 |
| swills_treat | -2.2564 | 0.9077 | -2.4859 | 0.0129 | 0.1047 | 0.0141 | 0.5372 |
| years_current_herd | 0.4653 | 0.3355 | 1.3869 | 0.1655 | 1.5925 | 0.8838 | 3.3947 |
| aware_control_measure | 1.2552 | 0.8058 | 1.5576 | 0.1193 | 3.5085 | 0.7547 | 19.1716 |
| protective_gear | 0.0010 | 0.8285 | 0.0012 | 0.9990 | 1.0010 | 0.1859 | 5.0714 |
| kitchen_leftover_pig_meat | 1.4507 | 0.8088 | 1.7935 | 0.0729 | 4.2660 | 0.9408 | 24.0469 |
| ticks_pigs_premises | 1.4009 | 0.9370 | 1.4950 | 0.1349 | 4.0588 | 0.7023 | 30.3487 |
