# Supplementary material for: African Swine Fever Perception, Risk Factors, and Socioeconomic Disparities Among Smallholder Domestic Pig Farmers in Serengeti, Tanzania
Source: Transbound Emerg Dis. 2025 Aug 27;2025:3922067. doi: 10.1155/tbed/3922067 (PMC12408130; doi:10.1155/tbed/3922067)
Supplement: Supporting Information 4 — Table 4: Multicollinearity test of Model 2 showing the variance inflation factor (VIF) for each variable. [file 3922067.f4.docx]

**Table 4:** Malticolinearity Test of Model 2 showing the Variance Inflation Factor (VIF) for each variable

| **Variable** | **VIF** |
| --- | --- |
| ASF encounter before | 1.2037 |
| Sold pig product with ASF before | 1.7978 |
| No action to prevent loss | 1.1900 |
| Years of domestic pig keeping | 1.4042 |
| Swills treat | 1.8071 |
| Years current herd | 1.3122 |
| Aware control measure | 1.2532 |
| Protective gear | 1.4993 |
| Kitchen leftover pig meat | 1.4288 |
| Ticks pigs premises | 1.7024 |

*Note: Base on VIF (i.e, variance inflation factor), the model does not have problem of multicollinearity. with all VIF less than 5 (James et al., 2023).
